# Registering prostate external beam radiotherapy with a boost from high-dose-rate brachytherapy: a comparative evaluation of deformable registration algorithms

**DOI:** 10.1186/s13014-015-0563-9

**Published:** 2015-12-14

**Authors:** Calyn R. Moulton, Michael J. House, Victoria Lye, Colin I. Tang, Michele Krawiec, David J. Joseph, James W. Denham, Martin A. Ebert

**Affiliations:** School of Physics (M013), University of Western Australia, 35 Stirling Highway, Crawley, Western Australia, 6009 Australia; Radiation Oncology, Sir Charles Gairdner Hospital, Hospital Avenue, Nedlands, Western Australia, 6009 Australia; School of Surgery, University of Western Australia, 35 Stirling Highway, Crawley, Western Australia, 6009 Australia; School of Medicine and Population Health, University of Newcastle, University Drive, Callaghan, New South Wales, 2308 Australia

**Keywords:** Contour propagation, Image similarity, Registration validation

## Abstract

**Background:**

Registering CTs for patients receiving external beam radiotherapy (EBRT) with a boost dose from high-dose-rate brachytherapy (HDR) can be challenging due to considerable image discrepancies (e.g. rectal fillings, HDR needles, HDR artefacts and HDR rectal packing materials). This study is the first to comparatively evaluate image processing and registration methods used to register the rectums in EBRT and HDR CTs of prostate cancer patients. The focus is on the rectum due to planned future analysis of rectal dose-volume response.

**Methods:**

For 64 patients, the EBRT CT was retrospectively registered to the HDR CT with rigid registration and non-rigid registration methods in VelocityAI. Image processing was undertaken on the HDR CT and the rigidly-registered EBRT CT to reduce the impact of discriminating features on alternative non-rigid registration methods applied in the software suite for Deformable Image Registration and Adaptive Radiotherapy Research (DIRART) using the Horn-Schunck optical flow and Demons algorithms. The propagated EBRT-rectum structures were compared with the HDR structure using the Dice similarity coefficient (DSC), Hausdorff distance (HD) and average surface distance (ASD). The image similarity was compared using mutual information (MI) and root mean squared error (MSE). The displacement vector field was assessed via the Jacobian determinant (JAC). The post-registration alignments of rectums for 21 patients were visually assessed.

**Results:**

The greatest improvement in the median DSC relative to the rigid registration result was 35 % for the Horn-Schunck algorithm with image processing. This algorithm also provided the best ASD results. The VelocityAI algorithms provided superior HD, MI, MSE and JAC results. The visual assessment indicated that the rigid plus deformable multi-pass method within VelocityAI resulted in the best rectum alignment.

**Conclusions:**

The DSC, ASD and HD improved significantly relative to the rigid registration result if image processing was applied prior to DIRART non-rigid registrations, whereas VelocityAI without image processing provided significant improvements. Reliance on a single rectum structure-correspondence metric would have been misleading as the metrics were inconsistent with one another and visual assessments. It was important to calculate metrics for a restricted region covering the organ of interest. Overall, VelocityAI generated the best registrations for the rectum according to the visual assessment, HD, MI, MSE and JAC results.

**Electronic supplementary material:**

The online version of this article (doi:10.1186/s13014-015-0563-9) contains supplementary material, which is available to authorized users.

## Introduction

Radiotherapy dose-volume parameters for specific organs have been associated with normal tissue toxicity [1]. However, the correlation between planned dose-volume parameters and observed toxicities is confounded by how well the planned dose reflects the dose delivered [2]. Hence, studies have focused on developing methods for accumulating dose from daily fractions [3] or combined treatments [4, 5].

Therapies with different fractionation can be adjusted for fractionation effects by converting to equieffective dose given in 2 Gy fractions (EQD 2_*α*/*β*_) [4, 6]. However, the anatomy in CTs may not coincide due to motion and variations in reference coordinate systems. Consequently, a ‘worst case’ assumption that the same volumes will receive the high doses is not necessarily valid as it is possible that a volume planned to receive a specific dose from one component could receive a different dose after adjustments for motion [7]. A rigid registration is not sufficient as non-rigid registration, also called deformable image registration (DIR), is required due to deformations and shrinkage [5]. A total dose distribution could be obtained after DIR by performing voxel-by-voxel summation of the EQD 2_*α*/*β*_ doses [4, 8]. Combining dose without applying DIR via post-planning the brachytherapy on the external beam radiotherapy (EBRT) planning CT has been explored [9] and is subject to whether post-planning the brachytherapy dose is adequate given anatomy changes.

The accuracies of DIR algorithms have been examined experimentally using deformed phantoms or image modification to include deformations [10, 11]. The reliability of DIR has been examined for each patient by checking the agreement between the manually-delineated structure for one CT and the DIR applied to the manually-delineated structure from the other CT [12, 13]. Clinical checks of the post-registration anatomical alignment can also be used [14, 15]. Additionally, metrics assessing the displacement vector field (DVF) and the similarity between one image and the deformed image have been proposed as tools for assessing the reliability of DIR [16]. One evaluation type may be more appropriate in certain situations [12, 16]. The deformed dose distribution can be used reliably when DIR is considered to be adequate [17].

Publications are lacking in the context of registering an EBRT pelvic CT to a high-dose-rate brachytherapy (HDR) pelvic CT. Image-intensity based DIR algorithms applied to such CTs are susceptible to errors when there are major image differences [18]. This application is problematic given that the time between the HDR and EBRT planning CTs can be months. The discrepancies between the CTs include varying amounts of bowel gas, rectal filling and general artefacts. Additionally, only the HDR CT contains the HDR needles, streak artefacts off the needles, low CT number pixels around the needles and rectal packing materials.

This study examines the performance of image processing and non-rigid registration tasks available in commercial software and customizations to an open-source package when applied to register the rectums in prostate EBRT and HDR data. Specifically, how did they perform in terms of the Dice similarity coefficient [12], Hausdorff distance [12], average surface distance [12], root mean squared error [12], mutual information [19], Jacobian determinant [12] and visual assessment [14]? We focus on the rectum due to planned future analysis of rectal dose-volume response for combined EBRT/HDR prostate treatment.

## Patient data

This study used treatment plans for 64 prostate cancer patients who were treated with EBRT followed by a boost dose from Iridium-192 HDR via after-loading hollow metal needles at Sir Charles Gairdner Hospital in the period 2004–2008. Patient criteria and treatment methodology were as specified for the Trans-Tasman Radiation Oncology Group (TROG) 03.04 Randomized Androgen Deprivation and Radiotherapy (RADAR) trial [20, 21]. A planning CT was acquired at the start of each treatment component (e.g. Additional file 1: Figures A1 and A2). The number of slices (EBRT 32–77, HDR 32–59) and the voxel spacing (EBRT 0.809–0.977 mm, HDR 0.242–0.566 mm) for the CTs varied; however, there was a common slice thickness (3 mm) and dimension (512 by 512 pixels).

The external wall of the rectum was manually delineated by treating clinicians in the EBRT CTs using the Elekta Focal treatment planning software (Elekta AB, Stockholm, Sweden) and in the HDR CTs using the Brachyvision planning software (Varian Medical Systems, Palo Alto, US). Rectum outlines were reviewed (by author MK) for consistency between patients. The superior border of the rectum structures in the EBRT CTs were defined by the level that the rectum turns horizontally into the sigmoid colon and the inferior border defined on the most inferior axial image slice on which the ischial tuberosities were visible. Any further references to rectum ‘structure’ refer to the 3D manual outline of the external rectum wall while ‘contour’ refers to the 2D section of this outline on a particular image slice.

### Ethics approval and consent to participate

The TROG 03.04 RADAR Trial is registered with the National Institutes of Health Clinical Trials Registry (number NCT00193856). This trial has approval from the Hunter New England Human Research Ethics Committee (Trial ID. 03/06/11/3.02), the Sir Charles Gairdner Group Human Research Ethics Committee (2003-050) and the University of Western Australia Human Research Ethics Office (RA/4/1/5601). Patients participating in the trial signed consent forms.

### Consent for publication

The signed patient consent forms for the trial informed patients that their medical information may be used to publish the results of the study. In accordance with the signed patient consent forms, this publication includes only anonymized information and does not include information identifying any patient.

## Methods

**Fig. 1:**
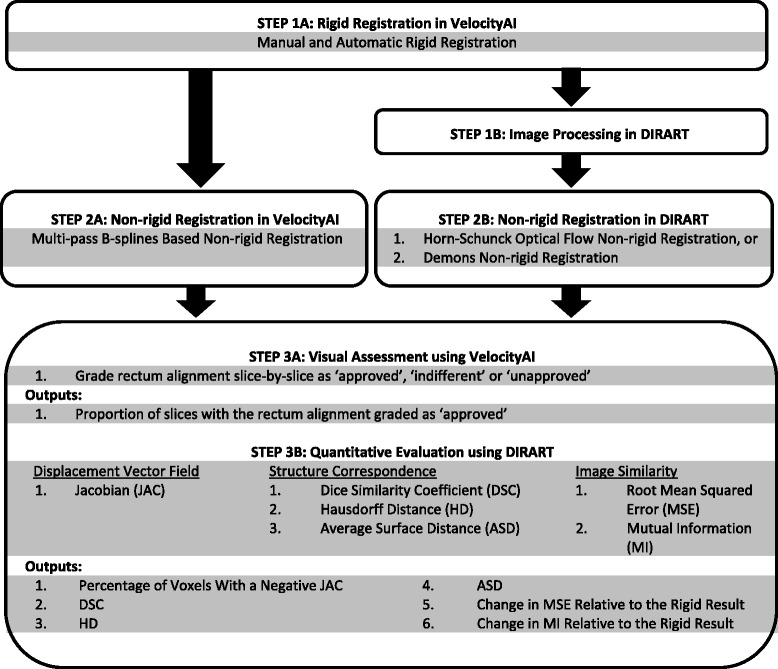
A summary of the image processing, registration and evaluation process. See the abbreviations list or the non-rigid registration section of the methods section for information about D, HS, V1 and V2

Figure 1 illustrates the registration and evaluation process detailed in this section.

### Rigid registration

A manual rigid registration (global translations and rotations) was performed in Velocity Advanced Imaging 2.8.1 (Varian Medical Systems, Palo Alto, US) to align the bony anatomy in the EBRT and HDR planning CTs. An automatic rigid registration was then performed to optimize the registration.

Copies of the HDR CT, the re-sampled rigidly-registered EBRT CT and the rectum structures from the HDR and rigidly-registered EBRT CTs were exported from Velocity Advanced Imaging (VelocityAI) in DICOM format for further image preprocessing in MATLAB^TM^ R2010a (The MathWorks Inc., Massachusetts, US), CERR (version 4.1) [22] and DIRART (version 1.0a) [23]. At the time of export the rigidly-registered EBRT CTs were re-sampled to have the same voxel sizes and dimensions as the HDR CTs (see earlier section on patient data), which covered a smaller field-of-view.

### Image preprocessing

Prior to DIR in DIRART the image processing detailed below was applied as the image processing led to a considerably improved post-registration rectum alignment. In Additional file 1: Figures A3 and A4 provide examples of slices of the final HDR and rigidly-registered EBRT CTs after image processing. The image processing steps are explained in detail in section I of Additional file 1. They key components are:

1. The HDR needles, HDR rectum packing material and HDR rectum low CT number artefacts were replaced with the average CT number of neighboring tissue pixels.
2. A Gaussian smoothing and blurring process was applied to avoid features in the HDR image caused by the previous pixel adjustments.
3. Rectum painting [14] with a uniform high CT number (2500) was applied to the final HDR and rigidly-registered EBRT CTs.

### Non-rigid registration (deformable image registration)

Image processing was not applied prior to DIR in VelocityAI as the post-registration alignment in VelocityAI was reasonable relative to registrations obtained in DIRART without image processing. The multi-pass DIRs in VelocityAI (version 2.8.1) were based on the B-spline algorithm with the Mattes mutual information metric [24]. Additionally, non-rigid registrations in VelocityAI were performed by applying a global scale registration immediately before DIR. The VelocityAI methods were rigid, rigid plus multi-pass DIR (V1) and rigid plus scale plus multi-pass DIR (V2).

The DIR in DIRART was applied to the EBRT rigidly-registered CT and the HDR CT after image processing as this led to a considerably-improved post-registration rectum alignment and made it more comparable with the VelocityAI alignments. The original Demons and original Horn-Schunck optical flow (HSOF) algorithms were used. These DIRs use the root of the mean of the squared-intensity differences as the image-similarity metric [23]. The default settings in DIRART were used [23, 24]. The image processing and DIRs applied in DIRART were rigid plus image-processing plus HSOF-DIR (HS) and rigid plus image-processing plus Demons-DIR (D).

### Evaluation

#### Visual assessments

The anatomical alignment for 64 patients was initially inspected by the researcher running each registration (author CRM). The post-DIR anatomical alignments for 21 of the 64 patients were inspected by a combination of in-training (author VL) and experienced (author CIT) radiation oncologists. The alignment between the rectums in the HDR CT and the registered EBRT CT was graded slice-by-slice using the spyglass tool in VelocityAI. The grades were ‘approved’, ‘indifferent’ or ‘unapproved’. The grading was based on whether the misalignment was clinically relevant and was similar to the situation where an observer has to decide if a contour is sufficiently inconsistent with anatomy to warrant re-contouring. The results were assessed by calculating the proportion of slices with grades of the ‘approved’ type.

#### Structure-correspondence metrics

The Dice similarity coefficient (DSC) was calculated as the volume of overlap of the two structures and normalized by the average volume of the structures. The DSC range is zero (no overlap) to one (perfect overlap) [12]. The Hausdorff distance (HD) was calculated as the maximum of the distances from a point on one 3D structure to the closest point on the other 3D structure [12]. The average surface distance (ASD) was calculated as the average of the distances from a point on one 3D structure to the closest point on the other 3D structure [12]. Due to considerable differences in the slice span of the rectum structures for the HDR and EBRT CTs, these metrics were calculated over slices where the HDR (fixed image) rectum structure existed.

#### Image-similarity metrics

Image similarity was examined via the percentage increase (decrease) in the image-similarity (dissimilarity) metric relative to that before the registration. The mutual information (MI) was used for similarity and the root of the mean squared error (MSE) for dissimilarity [12, 19]. Using these two metrics ensured assessment with at least one image-similarity metric that was different to the metric used in the DIR algorithm to optimize the registration.

#### Displacement-vector-field metric

Physically unachievable organ deformations are indicated by negative Jacobian determinants (JAC) of the DVF [12]. Consequently, the physically-unachievable characteristics of the DVF can be summarized via the percentage of voxels with a negative JAC.

### Statistical analysis

Paired percentage differences between the absolute DSC/ASD/HD results for different registration comparisons were tested for significance via exact Wilcoxon signed-rank tests against a zero median. The percentage JAC metric and the proportion of approved rectum-alignments for different registration comparisons were expressed in absolute difference and subject to the same test. Quantile-quantile plots showed that differences were not normally distributed. The tests were performed in R (version 2.15.2) [25] using the Coin package [26] and the Pratt method for zeros [27]. *P*-values were considered significant if less than 0.05.

## Results

### Visual assessments

The major misalignments after DIR were observed around the pubic symphysis, ischium near the inferior extent of the obturator foramen, superior ramus of pubis near the obturator canal, coccyx, medial aspect of the acetabulum and anterior side of the rectum (see Additional file 1: Figure A5 for labeling of anatomy).

The medians of pairwise differences in the proportions of slices with the alignment of the rectum approved for various DIR comparisons of the V1, V2, D and HS methods are provided in Table 1. According to the median differences in rectum approval-proportions between registrations, the most useful to least useful alignments came from the V1, V2 and D/HS methods respectively. The median approval-proportions for the V1, V2, D and HS methods were 0.626, 0.574, 0.385 and 0.385 respectively.

**Table 1:** Registration comparisons via pairwise differences in the proportions of slices with the rectum alignment approved. See the abbreviations list or the non-rigid registration section of the methods section for information about D, HS, V1 and V2

		Rectum approval-proportion		
Registration comparison	*N*	median difference	*Z*-value	*P*-value
V2 versus V1	21	–0.032	–3.44	0.0002
HS versus V1	21	–0.169	–3.84	<0.0001
HS versus V2	21	–0.124	–3.22	0.0006
D versus V1	21	–0.241	–4.02	<0.0001
D versus V2	21	–0.189	–4.00	<0.0001
D versus HS	21	0	–	–

*N* is the number of patients to whom the registrations were applied. The pairwise difference is calculated as the first mentioned registration subtract the second mentioned registration. The *Z*-value and *p*-value are from the Wilcoxon signed-rank test of the pairwise differences against a median of zero (this test is not appropriate when the median difference is zero). A significant negative difference indicates the second mentioned registration is superior

The registration package providing the best rectum registration according to the other metrics detailed in the following sections was consistent irrespective of whether the metrics were calculated for the 64 patients or the subsample used for the visual assessments (see Additional file 2 for the results when metrics are calculated for the subsample). Consequently, the results for the metrics when they were calculated across the full analyzed data set were compared with the visual assessment results.

### Structure-correspondence metrics

**Fig. 2:**
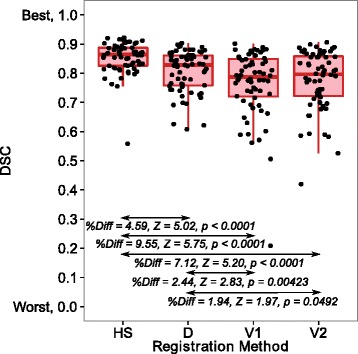
The Dice similarity coefficient (DSC) results for registrations applied to 64 patients. The figure includes the median (thick horizontal line), interquartile ranges (large boxes), maximums/minimums without outliers (vertical lines from large boxes) and raw data points (filled circles). The median pairwise percentage difference (%Diff) between the indicated registrations is provided alongside the *Z*-values (Z) and *p*-values (p) from exact Wilcoxon signed-rank tests of a median of zero for the pairwise percentage difference. The difference was calculated as the registration on the left subtract the registration on the right and this difference was expressed as a percentage of the registration on the right. A significant positive percentage difference in DSC indicates that the registration on the left is superior. See the abbreviations list or the non-rigid registration section of the methods section for information about D, HS, V1 and V2

Figure 2 shows the DSCs after the HS, D, V1 and V2 methods for the 64 patients. Additionally, the median and interquartile range for the rigid registration DSCs were 0.641 and 0.142. The medians of the percentage differences between the DSC results for most comparisons of the rigid, V1, V2, D and HS registrations were significantly different from zero given the Wilcoxon test *Z*-values and *p*-values. The significant differences for the HS, D, V1 and V2 registration comparisons are indicated in Fig. 2. The HS method achieved the best DSC results in terms of percentage differences with the other methods (Fig. 2).

**Fig. 3:**
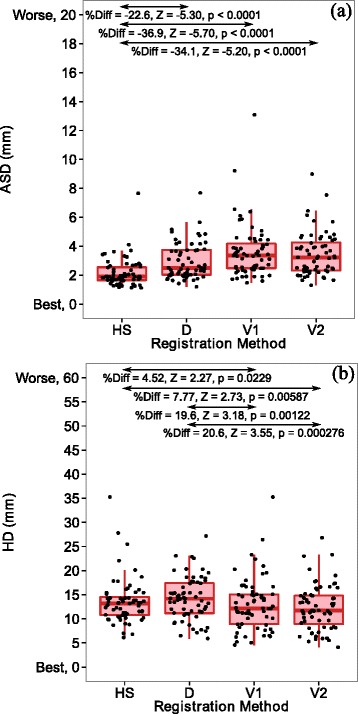
**a** Average surface distance (ASD) and **b** Hausdorff (HD) results for registrations applied to 64 patients. The figures include the median (thick horizontal line), interquartile ranges (large boxes), maximums/minimums without outliers (vertical lines from large boxes) and raw data points (filled circles). The median pairwise percentage difference (%Diff) between the indicated registrations is provided alongside the *Z*-values (Z) and *p*-values (p) from exact Wilcoxon signed-rank tests of a median of zero for the pairwise percentage difference. The difference was calculated as the registration on the left subtract the registration on the right and this difference was expressed as a percentage of the registration on the right. A significant negative percentage difference in ASD indicates that the registration on the left is superior. A significant positive percentage difference in HD indicates that the registration on the right is superior. See the abbreviations list or the non-rigid registration section of the methods section for information about D, HS, V1 and V2

Figure 3a and b show the ASD and HD results after the HS, D, V1 and V2 registration methods for the 64 patients. The significant differences for the HS, D, V1 and V2 registration comparisons via Wilcoxon signed-rank tests on pairwise percentage differences are indicated in Fig. 3a and b. The ASDs for the HS method were significantly smaller (smaller average shape discrepancy) than those for the D, V1 and V2 methods (Fig. 3a). However, the HDs for the V1 and V2 methods were significantly smaller (smaller extreme shape discrepancy) than those for HS and D methods (Fig. 3b).

All non-rigid registration methods led to a significant percentage improvement of the DSC, ASD and HD from the rigid registration result (see Additional file 1: Table A1 for statistical results).

### Image-similarity metrics

**Fig. 4:**
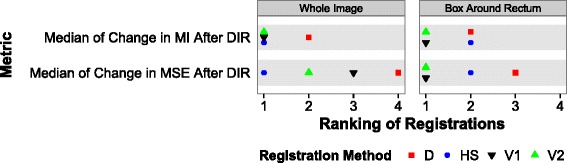
Ranking of registration methods according to image-similarity results for registrations applied to 64 patients. The medians of the percentage changes in the mean square error (MSE) and mutual information (MI) were calculated via 100*after/before-100. Increasing ranking indicates less image similarity and more image dissimilarity. Some registrations share the same ranking due to insignificant (*p* >0.05) paired differences for metric values (alternatively, see Additional file 1: Figures A6 and A7 for metric values and the results of the statistical significance tests). The MSE and MI metrics were calculated for two regions of interest, which were the entirety of the images and the bounding box enclosing both the HDR CT and rigidly-registered EBRT CT rectum structures. See the abbreviations list or the non-rigid registration section of the methods section for information about D, HS, V1 and V2

Figure 4 summarizes the image similarity results by ranking the V1, V2, D and HS methods according to the MI and MSE values for the 64 patients (alternatively, see Additional file 1: Figure A6 for the values). The registrations with insignificant pairwise differences in metric values according to Wilcoxon signed-rank tests were assigned the same ranking in Fig. 4. Alternatively, see Additional file 1: Figure A7, Tables A2 and A4 for the statistical results.

Considering similarity over the entire images, the HS method led to the best change (greatest percentage reduction) in the median MSE relative to the rigid registration value (Fig. 4), whereas the HS/V1/V2 methods inseparably led to the best change (greatest percentage increase) in the median MI for similarity over the entire images (Fig. 4). However, the V1 and V2 methods inseparably provided the best changes in the median MSE and median MI when considering similarity within the 3D bounding box enclosing both the HDR CT and rigidly-registered EBRT CT rectum structures (Fig. 4). For the DIRART methods, the MI decreased (deteriorated) relative to the rigid registration result and the MSE increased (deteriorated) relative to the rigid result when considering similarity in the 3D bounding box (Additional file 1: Figure A6).

### Displacement-vector-field metrics

To determine orderings, Wilcoxon signed-rank tests of the pairwise differences in the percentage of voxels with a negative JAC between the HS, D, V1 and V2 methods were performed for the 64 patients. When the V2, V1, HS and D methods were compared for the DVF across the whole image, the ordering of methods according to increasing percentage of voxels with a negative JAC was D, V2 and V1/HS. However, the medians of the percentages of voxels with a negative JAC were zero for the VelocityAI methods when calculations were restricted to the region contained by the volume of the rigidly-registered EBRT rectum structure. For this region, the ordering of registrations in terms of increasing percentages of voxels with a negative JAC was V1/V2, D and HS. Alternatively, Additional file 1: Figure A6 provides values with the test results detailed in Figure A7, Tables A2 and A3.

## Discussion

### Visual assessments were important

The V1 method was superior to the V2, HS and D methods in terms of significant differences in the proportions of slices with the rectum-alignment approved according to the visual assessments. Additionally, the VelocityAI methods (V1 and V2) resulted in superior rectum alignment approval-proportions compared to the DIRART methods. This was inconsistent with the structure-correspondence metric results, where the HS and D methods achieved better DSCs with worse HDs. The inconsistency between the results of metrics and visual assessments has been identified before [28]. Additionally, in this case it supports the current practice that a sole structure-correspondence metric cannot be used for the remaining registrations of the larger dataset as a filtering measure in lieu of a slice-by-slice visual assessment by expert observers.

The visual assessment results can be confounded by intra-observer and inter-observer assessment variations [28]; however, the impact of these variations was reduced by conducting the analysis via paired registration differences and the same observer assessing the four registrations per patient in a consecutive manner.

### Deformable image registration improved the rigid registration results

DIR was useful as, for example, the DSC, ASD and HD results were improved by applying DIR methods after rigid registration. The improvement in the median DSC was 35 % for the HS algorithm with image processing as compared to rigid registration. This compares well with the 31 % improvement in the mean DSC obtained by a study using the same algorithm with similar image processing tasks in the context of registering daily mega-voltage CT images to treatment planning kilo-voltage CT images [29]. The results and comparisons are confounded by inter/intra-observer variations in contouring [30].

### The choice of metrics and the way they were calculated were important

The results for the structure-correspondence metrics indicate that the selection of structure-correspondence metrics should be made carefully. The HS method was superior to the D, V1 and V2 methods in terms of a better structure-volume match (DSC) and less overall shape discrepancy (ASD). The V1 and V2 methods were superior to the HS and D methods in terms of extreme shape discrepancy (HD). The inconsistency of these metrics contrasts with another study where they were useful for evaluations [12]. In this case, the most extreme shape discrepancy (HD) is important from a dosimetric perspective as the anterior side of the registered rectum-structure could deviate from the fixed structure by extending over the brachytherapy high-dose area. Consequently, the correlation between the most extreme shape discrepancy and the high-dose parameters after registration may be useful when checking the validity of deformed dose.

It is important to calculate metrics over a restricted region that covers the area of concern or the organ at risk rather than the whole image when assessing whether the registration is acceptable in that area or for the organ at risk. The reason is that the registration is optimized over a region of interest and the performance can vary locally. For example, the V1/V2 methods provided optimal rectum results in terms of MI and MSE calculated in the region defined by the volume of the rigidly-registered EBRT rectum structure, whereas the HS method provided the best MSE result when calculated over the whole image. Additionally, unlike the DIRART algorithms the VelocityAI algorithms led to improvements in the image-similarity metrics calculated across the rectum relative to the rigid registration result. The choice of metrics can be important as elsewhere the MSE was found to not be useful for evaluation [12].

### The B-splines based registration resulted in the best registration of the rectum

The results for the rectum were sufficiently different to distinguish the best-performing VelocityAI registration from the best-performing DIRART registration. Relative to the DIRART algorithms, the VelocityAI algorithms did achieve better image similarity and visual alignment over the region contained by the volume of the rectum structure. Additionally, the VelocityAI algorithms appeared to do so with less physically-unrealistic displacements (smaller percentage of displacements with negative JACs) and less extreme shape discrepancy between the fixed and propagated rectum structures (smaller HD). As there was no image processing prior to the VelocityAI algorithms, the VelocityAI algorithms achieved these superior results whilst exposed to rectum discrepancies. As such, this study demonstrates the VelocityAI DIRs (B-splines based) appeared to result in the best rectum alignment and achieve DVFs with the least physically-unrealistic displacements.

This evaluation is based on the algorithms in the form they were released. Also, the user cannot change the registration parameters in VelocityAI. If the parameters in both packages were adjustable it would be a useful and difficult task to find optimal performance [24].

The comparative evaluations of rectum registrations from different registration systems is important for adequately accumulating dose for combined EBRT/HDR prostate cancer treatment and correlating it with observed gastrointestinal toxicities. The assessment of impact of image registration on dose-outcomes correlation will provide additional validation of the alternative approaches, and this is the subject of ongoing investigation.

### Recommendations and future considerations

- Registrations may benefit from images immediately prior to the HDR insertion of needles as this may allow changes over the preceding months to be separated from changes due to HDR needles and treatment positioning.
- Given the image discrepancies, it would be useful to evaluate registrations including a recently-developed penalty term minimizing the volume of missing information [18], methods that exclude the rectum discrepancies [31, 32] or changes to DIRART to use other image-similarity metrics (e.g. mutual information).
- Evaluation of registrations customized for the urethra, bladder, prostate and seminal vesicles would be useful as they require work on considerable image issues (e.g. HDR needles in the prostate and the urethra catheter balloon in the bladder).
- Registration evaluation for patients can be difficult and involve a variety of methods as there is no direct measure of registration error due to no known ground truth. Information obtained from other evaluation methods such as landmarks, phantoms and deformed dose uncertainty [24, 33–36] would be useful if applied to HDR CTs given the image contents.

## Conclusion

This study demonstrated that structure correspondence, image similarity and visual assessments are useful for assessing registrations applied to EBRT and HDR CTs of prostate cancer patients. We found that using non-rigid registrations in VelocityAI or image processing plus non-rigid registrations in DIRART improved the alignment of the rectum according to visual assessment and various metrics. It would have been misleading to use a structure-correspondence metric as a sole indicator of rectum alignment given that such metrics were inconsistent with other metrics and visual assessments. It is recommended that image-similarity and displacement-vector-field metrics be calculated for a restricted region covering the organ of interest instead of using global values. Applying the DIR methods in VelocityAI provided the most optimal registration result for the rectum as assessed by the greatest rectum alignment approval-proportion, the least extreme shape discrepancy between rectum structures and the most optimal rectum image similarity. We encourage the development of registrations for the prostate and urethra in EBRT and HDR CTs as doses to the prostate and urethra are key clinical concerns in the RADAR trial.

## Additional files

Additional file 1 **Online supplementary material providing additional method details, patient images, metric values and statistical analysis.** (PDF 0.46 kb)

Additional file 2 **Online supplementary material providing metric results for the 21 patient subsample.** (PDF 796 kb)

## Abbreviations

%Diff: median pairwise percentage difference
ASD: average surface distance
CERR: computational environment for radiotherapy research
D: rigid plus image processing plus Demons-DIR in DIRART
DIR: deformable image registration
DIRART: deformable image registration and adaptive radiotherapy research
DSC: dice similarity coefficient
DVF: displacement vector field
EBRT: external beam radiotherapy
EQD2: equieffective dose given in 2 Gy fractions
HD: Hausdorff distance
HDR: high-dose-rate brachytherapy
HS: rigid plus image processing plus HSOF-DIR in DIRART
HSOF: Horn-Schunck optical flow registration algorithm
JAC: Jacobian determinant
MI: mutual information
MSE: root of the mean squared error
p: *p*-value from Wilcoxon signed-rank test
RADAR: randomized androgen deprivation and radiotherapy
TROG: trans-tasman radiation oncology group
V1: rigid plus multi-pass DIR in VelocityAI
V2: rigid plus scale plus multi-pass DIR in VelocityAI
VelocityAI: velocity advanced imaging
Z: *z*-value from Wilcoxon signed-rank test
