# Supplementary material for: Registering prostate external beam radiotherapy with a boost from high-dose-rate brachytherapy: a comparative evaluation of deformable registration algorithms
Source: Radiat Oncol. 2015 Dec 14;10:254. doi: 10.1186/s13014-015-0563-9 (PMC4678702; doi:10.1186/s13014-015-0563-9)
Supplement: Supplementary file 1 — Online supplementary material providing additional method details, patient images, metric values and statistical analysis. (PDF 0.46 kb) [file 13014_2015_563_MOESM1_ESM.pdf]

Additional file 1: Online Supplementary Material 1

Calyn R Moulton* and Michael J House

School of Physics, University of Western Australia, Crawley, Western Australia.

Victoria Lye, Colin I Tang, Michele Krawiec, David J Joseph,† and Martin A Ebert‡

Radiation Oncology, Sir Charles Gairdner Hospital, Nedlands, Western Australia.

James W Denham

School of Medicine and Population Health,
University of Newcastle, Callaghan, New South Wales.

* calyn.moulton@research.uwa.edu.au
† School of Surgery, University of Western Australia, Crawley, Western Australia.
‡ School of Physics, University of Western Australia, Crawley, Western Australia.

I. IMAGE PROCESSING DETAILS

1. HDR needles were detected by thresholding CT numbers ($\geq$3500). For each axial slice, a boundary was extracted from the outer most detected points. It was empirically determined that dilating the boundary in each axial slice by 6 voxels radially allowed the entirety of the HDR needles and associated artefacts to be contained. The pixels in the interior of the new boundaries were checked for a CT number above that of muscle/tissue (1200) or below that of fat/tissue (800). The detected pixels were replaced with the average CT number of neighboring undetected pixels. A binary mask was created from these detected pixels. The Hounsfield number is the CT number minus 1000.

2. For the HDR CTs, the rectum packing material extended beyond the superior-inferior extent of the HDR rectum structure. As such, pixels beyond the superior-inferior extent of the HDR structure that contained rectum packing material ($\geq$1200) and low CT number artefacts ($\leq$800) were detected and replaced with the average CT number of neighboring undetected pixels. The earlier mentioned binary mask was updated to include these detected pixels.

3. The following Gaussian smoothing and blurring process resulted in balanced HDR CT ($I_{balanced}$) and was applied to avoid features in the image caused by the pixel adjustments in steps 1 and 2:

   (a) A smoothed image ($I_{smoothed}$) was obtained by passing the adjusted HDR image ($I_{adjusted}$) through a Gaussian low pass-filter ($\sigma = 2$ voxels).

   (b) A blurred binary mask ($M_{blurred}$) was obtained by applying a Gaussian low-pass filter ($\sigma = 2$ voxels) to the binary mask.

   (c) A balanced HDR image ($I_{balanced}$) was obtained from the adjusted image ($I_{adjusted}$), smoothed image ($I_{smoothed}$) and the blurred binary mask ($M_{blurred}$) according to (1).

$$I_{balanced} = I_{adjusted} \times (1 - M_{blurred}) + I_{smoothed} \times M_{blurred} \tag{1}$$

4. An approach for dealing with inconsistent contents between the rectum structures for the EBRT and HDR CTs is to paint the contents of the rectum structures with a uniform CT number. The contents of rectum structures were replaced with a high CT number (2500), which was empirically determined as separating the rectum from surrounding tissue. The final HDR and rigidly-registered EBRT CTs were obtained by applying this rectum-structure-painting to the balanced HDR CT ($I_{balanced}$) and the rigidly-registered EBRT CT.

II. FIGURES AND TABLES COVERING ADDITIONAL ANALYSIS

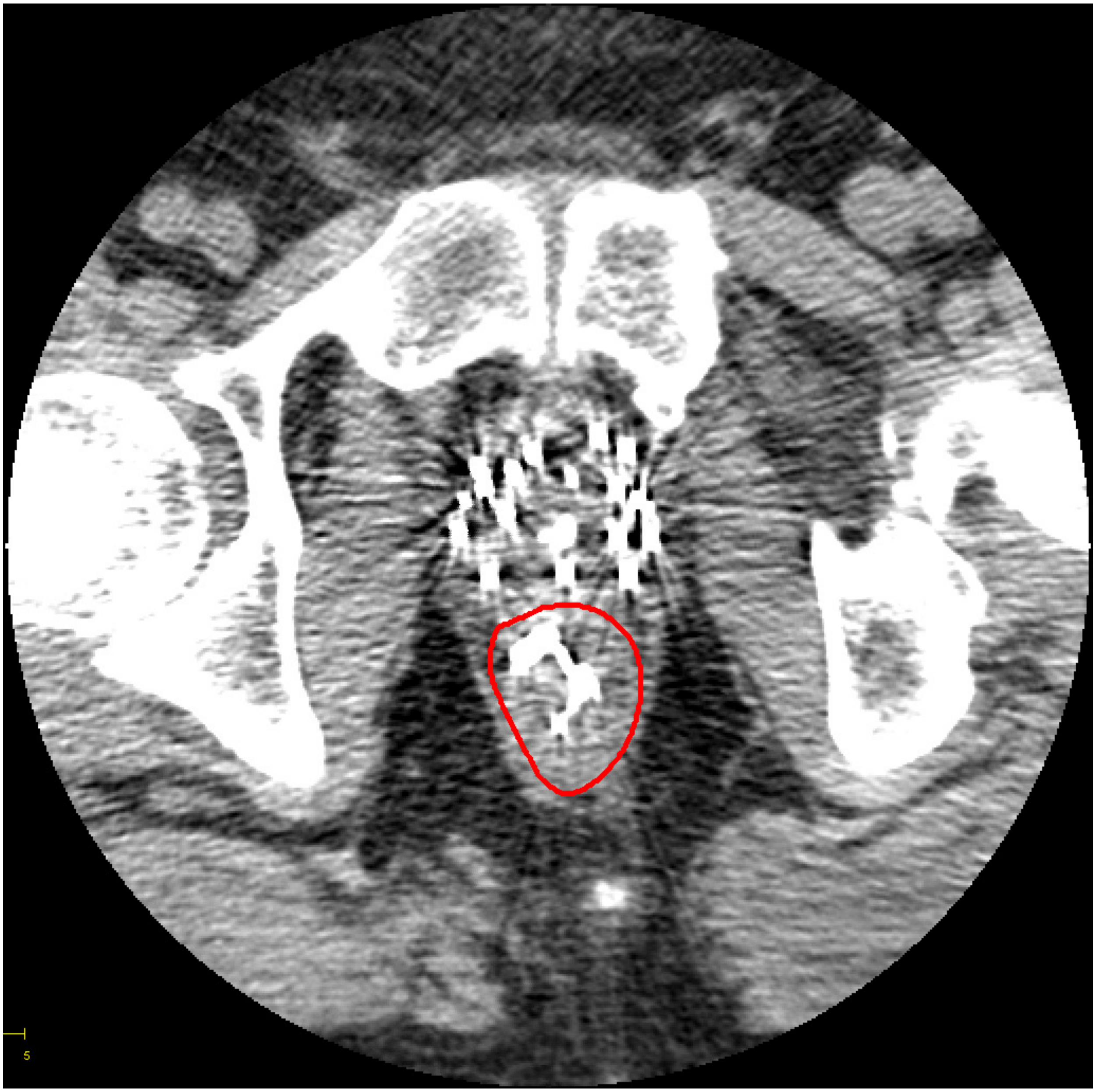

FIG. A1. A planning CT and rectum contour for high-dose-rate brachytherapy (HDR).

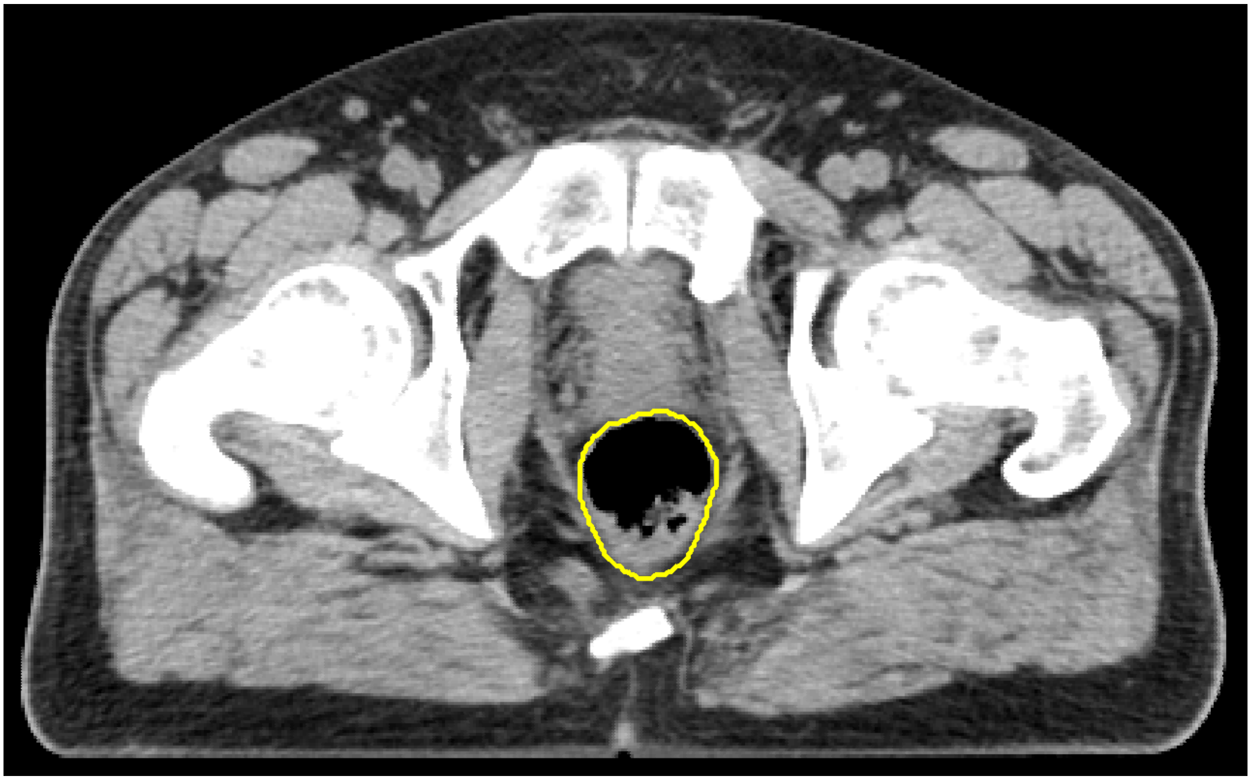

FIG. A2. A planning CT and rectum contour for external beam radiotherapy (EBRT).

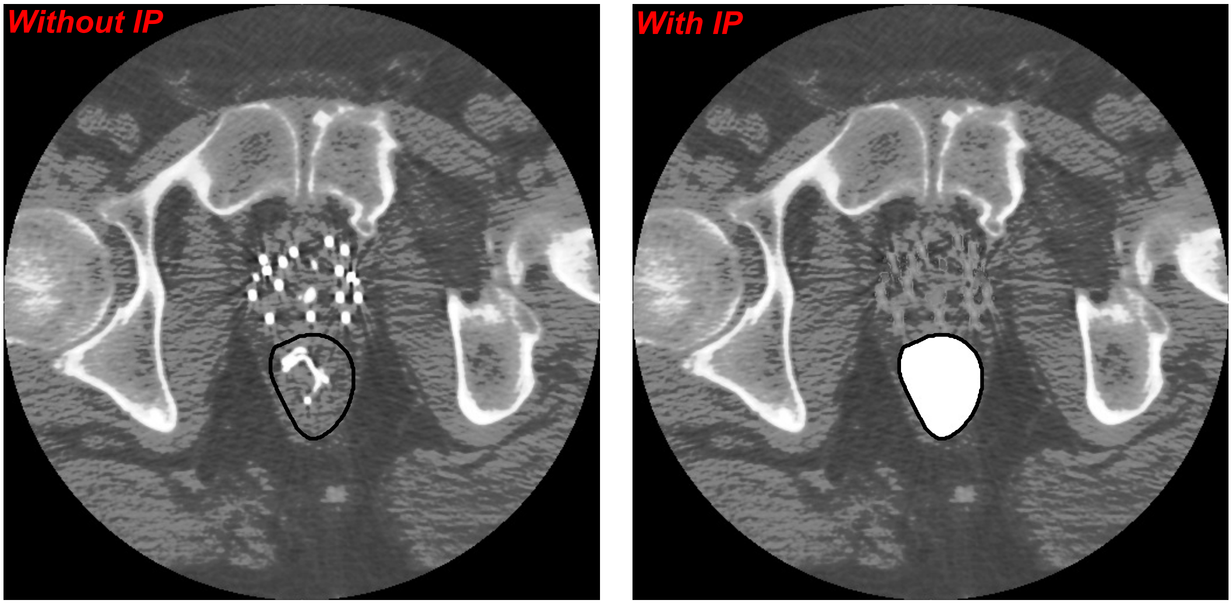

FIG. A3. The high-dose-rate brachytherapy (HDR) CT with and without image processing (IP). The contour is in black.

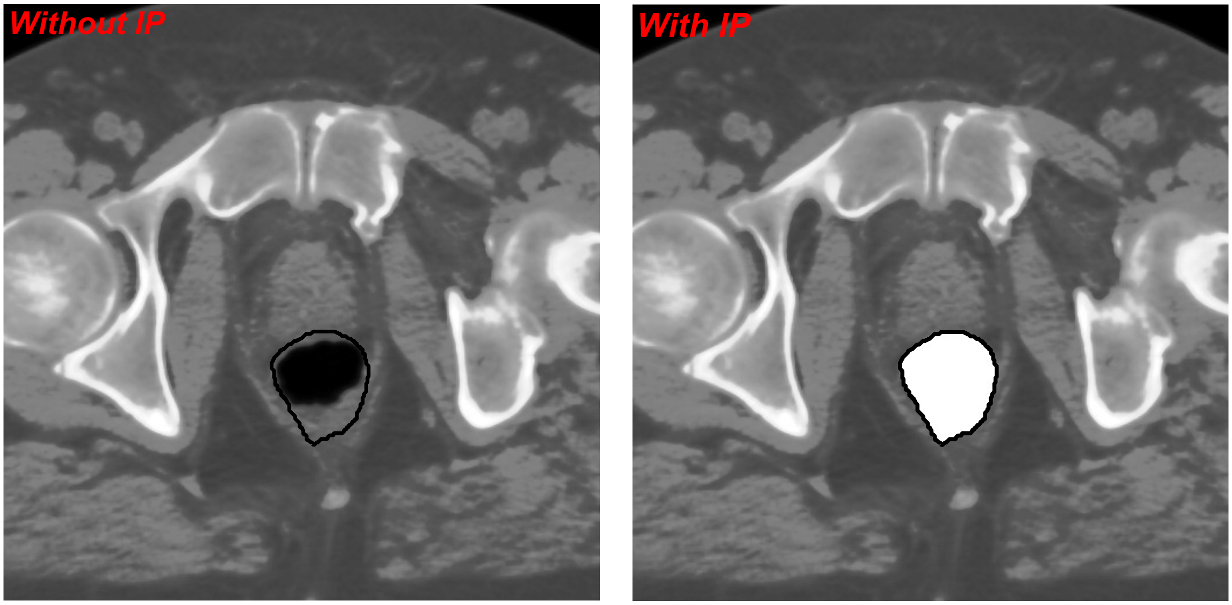

FIG. A4. The rigidly-registered external beam radiotherapy (EBRT) CT with and without image processing (IP). The contour is in black.

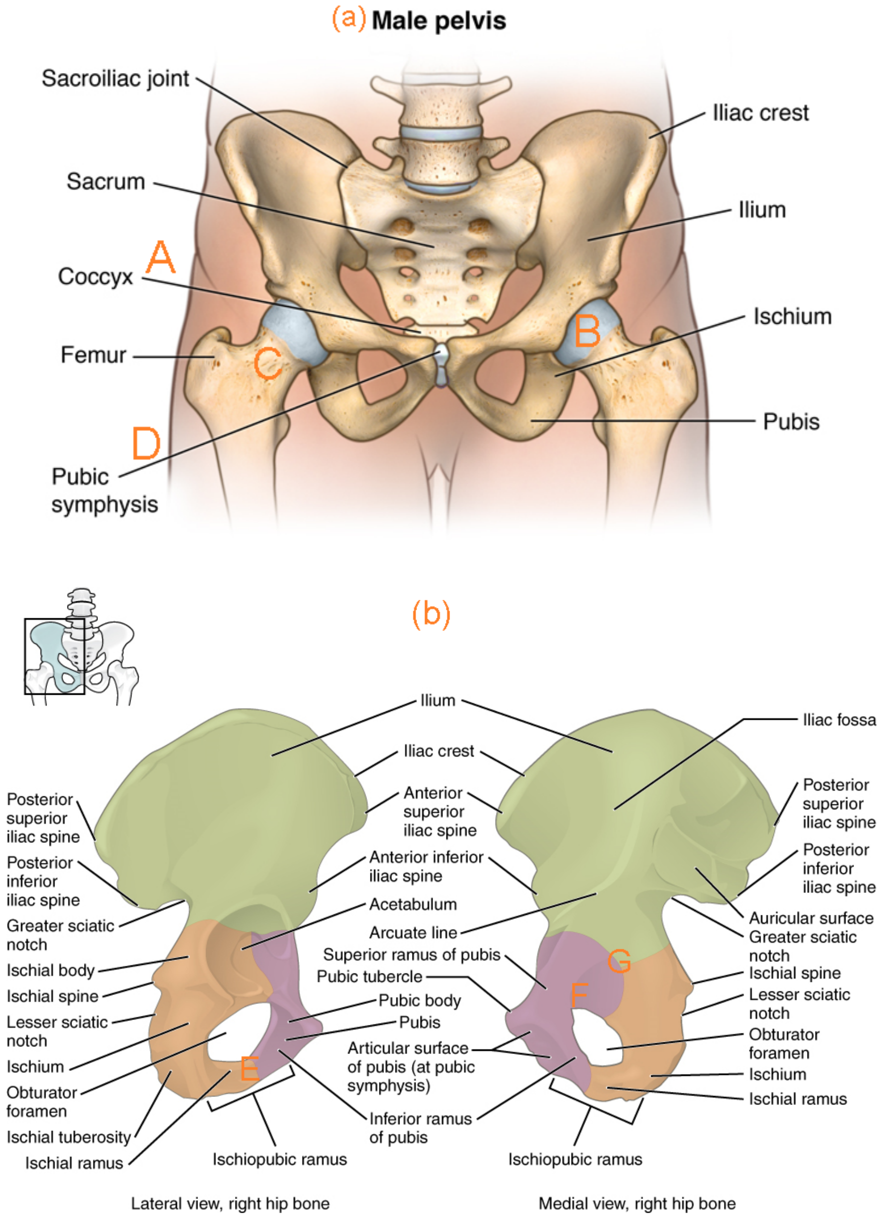

FIG. A5. (a) Male pelvis with the coccyx (A), femoral heads (B), femoral necks (C) and pubic symphysis (D) marked. [Source: University of Rochester. 2015. Anatomy of the Male and Female Pelvis. University of Rochester Medical Center - Online Medical Encyclopedia, June24, 2015. http://www.urmc.rochester.edu/Encyclopedia/GetImage.aspx?ImageID=322083] (b) Hip bone with the ischium near the inferior extent of the obturator foramen (E), superior ramus of pubis near the obturator canal (F) and medial aspect of the acetabulum (G) marked [Source: OpenStax College. 2013. The Pelvic Girdle and Pelvis. OpenStax-CNX, June 4, 2013. https://legacy.cnx.org/content/m46375/1.3/.]. The major anatomy misalignments for rigid registrations were observed around the coccyx, ischium near the inferior extent of the obturator foramen, superior ramus of pubis near the obturator canal, femoral heads and femoral necks. The rigid registration was observed in the majority of slices to provide unacceptable alignment of the rectum and soft tissue surrounding the rectum. After DIR, the major anatomy misalignments were observed around the pubic symphysis, ischium near the inferior extent of the obturator foramen, superior ramus of pubis near the obturator canal, coccyx and the medial aspect of the acetabulum.

TABLE A1. A summary of the improvement in the Dice similarity coefficient (DSC), average surface distance (ASD) and Hausdorff distance (HD) under various registration method comparisons for 64 patients via the median of percentage changes [X versus Y = 100*(X/Y-1)] and exact Wilcoxon singed-rank tests of medians of zero. A positive percentage difference in DSC indicates that the first mentioned registrations is superior as it had the larger DSC and a larger DSC indicates more contour overlap. A negative percentage difference in ASD indicates that the first mentioned registration is superior as it had the smaller ASD and a smaller ASD indicates closer overall contour shape matching. A negative percentage difference in HD indicates that the first mentioned registration is superior as it had the smaller HD and a smaller HD indicates a less extreme contour shape discrepancy.

	DSC Results			ASD Results			HD Results		
**Registration Comparison**	Median Change (%)	Signed-rank Z-value	Signed-rank P-value	Median Change (%)	Signed-rank Z-value	Signed-rank P-value	Median Change (%)	Signed-rank Z-value	Signed-rank P-value
V1 versus Rigid	19.4	6.94	**<0.0001**	-37.9	-6.96	**<0.0001**	-15.5	-6.53	**<0.0001**
V2 versus Rigid	22.6	6.94	**<0.0001**	-39.7	-6.95	**<0.0001**	-16.7	-6.57	**<0.0001**
D versus Rigid	26.8	6.87	**<0.0001**	-47.1	-6.77	**<0.0001**	-9.17	-2.37	**0.0170**
HS versus Rigid	32.0	6.96	**<0.0001**	-63.0	-6.96	**<0.0001**	-15.6	-5.58	**<0.0001**

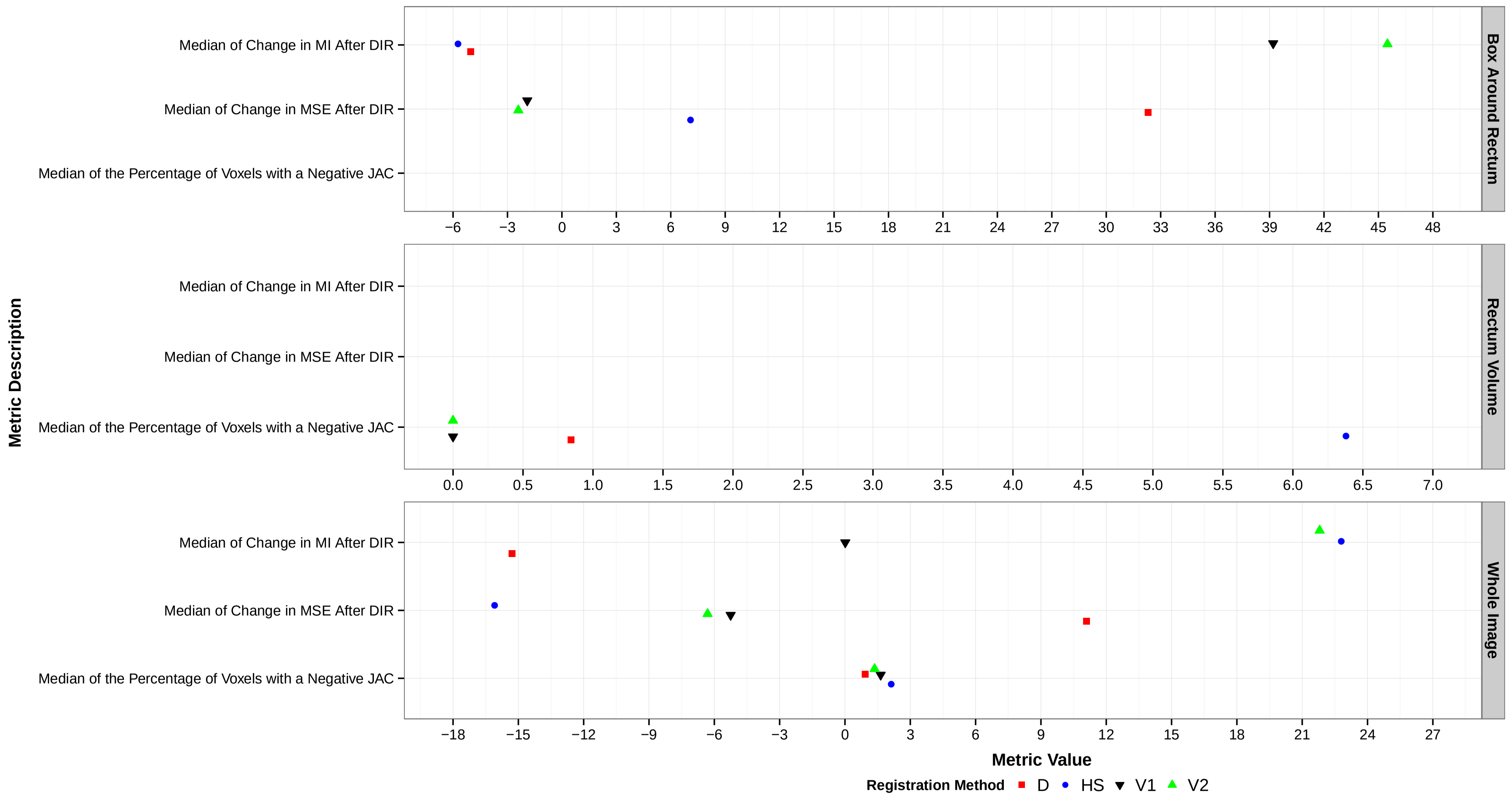

FIG. A6. The medians of the percentage of voxels with a negative JAC after various registration methods applied to 64 patients. Additionally, the medians of the percentage changes (e.g. $100\times$after/before $-$ 100) in the MSE and MI image-similarity metrics due to registrations applied to 64 patients. The percentage changes in the MSE and MI are the values after DIR relative to values after rigid registration. Consequently, a negative change in MI or a positive change in MSE indicate that the DIR has not improved upon the image similarity achieved by the rigid registration. For each metric you read horizontally to get the metric values for the various registrations indicated by symbols. The JACs were calculated across the entire displacement vector field and the region contained by the volume of the rigidly-registered EBRT rectum structure. The MSE and MI metrics were calculated for the entirety of the images and a bounding box enclosing both the HDR CT and rigidly-registered EBRT CT rectum structures.

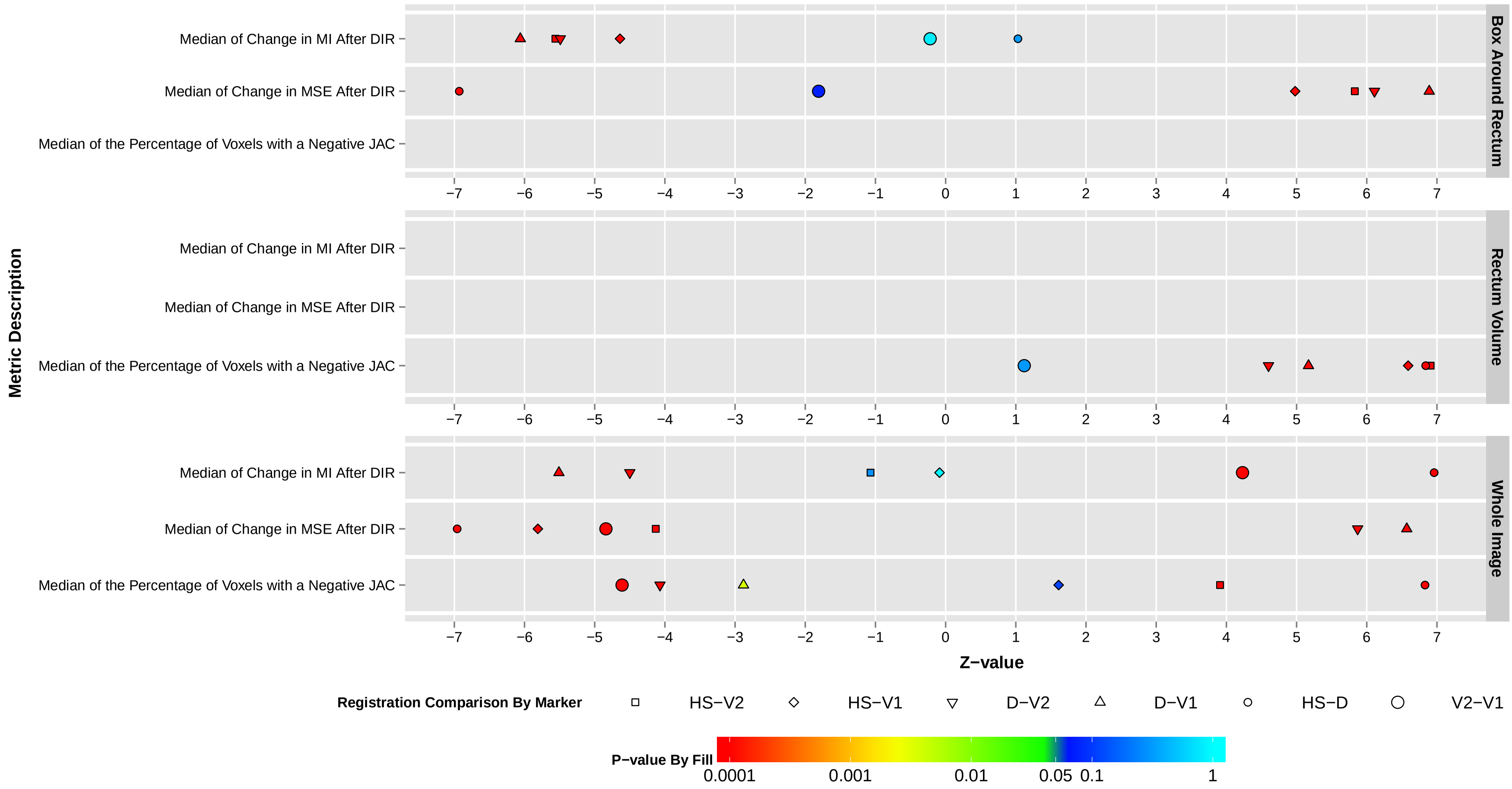

FIG. A7. The results for the exact Wilcoxon signed-rank test of the pairwise differences in the JAC, MSE and MI metric values between various registration methods applied to 64 patients. The JACs were calculated across the entire displacement vector field and the rigidly-registered EBRT CT rectum structure volume. The MSE and MI metrics were calculated for the entirety of the images and a bounding box enclosing both the HDR CT and rigidly-registered EBRT CT rectum structures. For each metric you read horizontally to get the Z-values for tests on the paired difference against a median of zero. The p-values associated with the Z-values are provided by a color fill of the plot markers.

Registration Comparison (Whole Image)		Metric		
		Median of the Percentage of Voxels with a Negative JAC	Median MSE Change After DIR (%)	Median MI Change After DIR (%)
HS3 - V2	M:	0.567	-0.097	0.00915
	Z:	3.91	-4.13	-1.07
	p:	0.0001	0.0001	0.288
HS3 - V1	M:	0.313	-0.116	0.154
	Z:	1.61	-5.81	-0.0869
	p:	0.110	0.0001	0.934
D3 - V2	M:	-0.288	0.185	-0.429
	Z:	-2.88	6.57	-5.51
	p:	0.00361	0.0001	0.0001
D3 - V1	M:	-0.668	0.169	-0.340
	Z:	-4.07	5.87	-4.50
	p:	0.0001	0.0001	0.0001
HS3 - D3	M:	1.19	-0.293	0.327
	Z:	6.83	-6.96	6.96
	p:	0.0001	0.0001	0.0001
V2 - V1	M:	-0.155	-0.0121	0.0684
	Z:	-4.61	-4.84	4.23
	p:	0.0001	0.0001	0.0001
Difference Method		Absolute	Absolute	Absolute

TABLE A2. Results for Wilcoxon signed-rank tests of the pairwise differences in JAC, MI and MSE metric values between the various registration methods applied to 64 patients. The JACs, MIs and MSEs were calculated across the whole image (entire displacement vector field). The paired metric comparison (difference method) is median absolute difference (M = X − Y) for metrics already in percentage format. The Z-values (Z) and p-values (p) from the tests that the medians are zero are included. A positive difference in the percentage of voxels with a negative JAC or change in MSE after DIR indicates that the second mentioned registration has less physical-unachievable displacements or less image dissimilarity respectively. A positive difference for the change in MI after DIR indicates that the first mentioned registration has superior image similarity.

Registration Comparison (Rectum Volume)		Metric: Median of the Percentage of Voxels with a Negative JAC
**HS3 - V2**	M:	5.84
	Z:	6.91
	p:	0.0001
**HS3 - V1**	M:	5.41
	Z:	6.59
	p:	0.0001
**D3 - V2**	M:	0.664
	Z:	5.17
	p:	0.0001
**D3 - V1**	M:	0.615
	Z:	4.60
	p:	0.0001
**HS3 - D3**	M:	4.53
	Z:	6.84
	p:	0.0001
**V2 - V1**	M:	0.0000
	Z:	1.12
	p:	0.300
**Difference Method**		Absolute

TABLE A3. Results for Wilcoxon signed-rank tests of the pairwise differences in the JAC metric values between the various registration methods applied to 64 patients. The JACs were calculated in the region contained by the volume of the rigidly-registered EBRT rectum structure. The paired metric comparison (difference method) is median absolute difference ($M = X - Y$) for metrics already in percentage format. The Z-values (Z) and p-values (p) from the tests that the medians are zero are included. A positive difference in the percentage of voxels with a negative JAC indicates that the second mentioned registration has less physical-unachievable displacements.

Registration Comparison (Box Around Rectum)		Metric	
		Median MSE Change After DIR (%)	Median MI Change After DIR (%)
HS3 - V2	M:	0.0932	-0.534
	Z:	5.83	-5.56
	p:	0.0001	0.0001
HS3 - V1	M:	0.105	-0.570
	Z:	4.98	-4.64
	p:	0.0001	0.0001
D3 - V2	M:	0.298	-0.603
	Z:	6.89	-6.06
	p:	0.0001	0.0001
D3 - V1	M:	0.313	-0.602
	Z:	6.11	-5.49
	p:	0.0001	0.0001
HS3 - D3	M:	-0.200	0.0511
	Z:	-6.93	1.03
	p:	0.0001	0.307
V2 - V1	M:	-0.00191	-0.00596
	Z:	-1.81	-0.221
	p:	0.0713	0.829
Difference Method		Absolute	Absolute

TABLE A4. Results for Wilcoxon signed-rank tests of the pairwise differences in the MI and MSE metric values between the various registration methods applied to 64 patients. The MIs and MSEs were calculated across a bounding box enclosing both the HDR CT and rigidly-registered EBRT CT rectum structures. The paired metric comparison (difference method) is median absolute difference ($M = X - Y$) as the metrics are already in percentage format. The Z-values (Z) and p-values (p) from the tests that the medians are zero are included. A positive difference in the change in MSE after DIR indicates that the second mentioned registration has less image dissimilarity. A positive difference for the change in MI after DIR indicates that the first mentioned registration has superior image similarity.
