# Supplementary material for: Registering prostate external beam radiotherapy with a boost from high-dose-rate brachytherapy: a comparative evaluation of deformable registration algorithms
Source: Radiat Oncol. 2015 Dec 14;10:254. doi: 10.1186/s13014-015-0563-9 (PMC4678702; doi:10.1186/s13014-015-0563-9)
Supplement: Supplementary file 2 — Online supplementary material providing metric results for the 21 patient subsample. (PDF 796 kb) [file 13014_2015_563_MOESM2_ESM.pdf]

# Additional file 2: Online Supplementary Material 2


Calyn R Moulton[*] and Michael J House

*School of Physics, University of Western Australia, Crawley, Western Australia.*

Victoria Lye, Colin I Tang, Michele Krawiec, David J Joseph,[†] and Martin A Ebert[‡]

*Radiation Oncology, Sir Charles Gairdner Hospital, Nedlands, Western Australia.*

James W Denham

*School of Medicine and Population Health,*
*University of Newcastle, Callaghan, New South Wales.*



[*] calyn.moulton@research.uwa.edu.au

[†] School of Surgery, University of Western Australia, Crawley, Western Australia.

[‡] School of Physics, University of Western Australia, Crawley, Western Australia.

TABLE B1. A summary of the registration comparison results provided in Tables B2 and B3 for 21 patients. The table includes which package (DIRART or VelocityAI) provided the best performing registration for the rectum according to different metrics. The metrics used to assess performance were the Dice similarity coefficient (DSC), average surface distance (ASD), Hausdorff distance (HD), root mean squared error (MSE), mutual information (MI) and Jacobian determinant (JAC) as calculated in Tables B2 and B3.

| **Metric Assessing Performance** | **Package Providing Best Registration** |
|---|---|
| DSC | DIRART |
| HD | VelocityAI |
| ASD | DIRART |
| MSE | VelocityAI |
| MI | VelocityAI |
| JAC | VelocityAI |

TABLE B2. A summary of the improvement in the Dice similarity coefficient (DSC), average surface distance (ASD) and Hausdorff distance (HD) under various registration method comparisons for 21 patients via the median of pairwise percentage changes [X versus Y = 100*(X/Y-1)]. A positive percentage difference in DSC indicates that the first mentioned registrations is superior as it had the larger DSC and a larger DSC indicates more contour overlap. A negative percentage difference in ASD indicates that the first mentioned registration is superior as it had the smaller ASD and a smaller ASD indicates closer overall contour shape matching. A negative percentage difference in HD indicates that the first mentioned registration is superior as it had the smaller HD and a smaller HD indicates a less extreme contour shape discrepancy.

| **Registration Comparison** | **DSC Results Median Change (%)** | **HD Results Median Change (%)** | **ASD Results Median Change (%)** |
|---|---|---|---|
| V1 versus Rigid | 19.9 | -14.1 | -34.0 |
| V2 versus Rigid | 20.0 | -12.6 | -32.5 |
| D versus Rigid | 24.5 | -1.15 | -46.5 |
| HS versus Rigid | 34.5 | -9.14 | -64.8 |
| HS versus D | 5.58 | -3.48 | -21.8 |
| HS versus V1 | 12.1 | 2.93 | -45.1 |
| HS versus V2 | 10.3 | 4.73 | -44.3 |
| D versus V1 | 2.85 | 19.0 | -13.5 |
| D versus V2 | 2.78 | 20.1 | -11.6 |

TABLE B3. A summary of the improvement in the root mean squared error (MSE), mutual information (MI) and Jacobian determinant (JAC) metrics under various registration method comparisons for 21 patients. The MSE and MI image-similarity metrics were calculated as the percentage changes in values from before and after DIR (e.g. $100\times\text{after}/\text{before} - 100$). The JAC metric was calculated as the percentage of voxels with a negative JAC. The reported medians are the median of the absolute pairwise difference between various registration methods [X versus Y = X-Y]. The MIs and MSEs were calculated across a bounding box enclosing both the HDR CT and rigidly-registered EBRT CT rectum structures. A positive (negative) difference in the change in MSE after DIR indicates that the second (first) mentioned registration has less image dissimilarity. A positive (negative) difference for the change in MI after DIR indicates that the first (second) mentioned registration has superior image similarity. The JACs were calculated in the region contained by the volume of the rigidly-registered EBRT rectum structure. A positive difference in the percentage of voxels with a negative JAC indicates that the second mentioned registration has less physical-unachievable displacements.

| **Registration** | **MSE Results** | **MI Results** | **JAC Results** |
|---|---|---|---|
| **Comparison** | **Median Change After DIR(%)** | **Median Change After DIR(%)** | **Median Percentage of JACs $< 0$ (%)** |
| HS versus D | -21.9 | 5.17 | 3.71 |
| HS versus V1 | 8.79 | -50.4 | 4.78 |
| HS versus V2 | 9.02 | -49.1 | 4.78 |
| D versus V1 | 26.5 | -54.1 | 0.625 |
| D versus V2 | 26.3 | -60.0 | 0.625 |
